# Influence of Filler Content and Filler Size on the Curing Kinetics of an Epoxy Resin

**DOI:** 10.3390/polym11111797

**Published:** 2019-11-01

**Authors:** Yang Zhao, Dietmar Drummer

**Affiliations:** Institute of Polymer Technology, University Erlangen-Nueremberg, 91058 Erlangen, Germany; yang.zhao@fau.de

**Keywords:** epoxy molding compound (EMC), reaction kinetics, filler content, filler size, isothermal DSC, Kamal-Sourour’s model, diffusion

## Abstract

In this research, the influences of filler content and filler particle size on the flow-hardening behavior were investigated by a measuring mixer. In order to more reliably assess the observed rheological behavior, isothermal differential scanning calorimetry (DSC) measurements were employed to study the curing kinetics of the compounds. The measured data can be fitted well with Kamal-Sourour’s model modified by the diffusion correlation according to Chern and Poehlein. After that, the influence of filler content and size on the kinetic parameters are presented discussed. The results show that the ultimate glass transition temperature is significantly lower for pure epoxy resin (EP) than for compounds filled with surface-treated glass beads, which have an essential effect on the diffusion-controlled reaction at different curing temperatures. For the surface-treated glass beads used in this study, the reaction speed in the early curing stage is accelerated by increasing filler content or decreasing of filler size. In the later curing stage, the reaction speeds of compounds with higher filler content or smaller fillers reduce more quickly. The study of reaction kinetics indicates that the activation energy *Ea*_1_, *Ea*_2_, the reaction order *m*, and *n* are affected differently by varying filler content and size.

## 1. Introduction

Recently, as the electrification of automobiles steadily advances, the cars today contain more and more electrical and electronic elements not only in the controlling system but also in the propulsion system. To ensure they protected from mechanical damage, contaminants, and moisture, electronics are often encapsulated in epoxy molding compounds (EMC) by using injection molding or transfer molding as manufacturing techniques for mass production [1]. These advanced applications demand outstanding material properties for the encapsulating material. In Epoxy resins, a three-dimensional network structure is present after curing, which leads to great mechanical and heat-/moisture-resistance properties, which are perfectly suitable to protect the devices from the environment. 

Similar to thermoplastics, these EMCs also contain different kinds of fillers to improve their mechanical, thermal or electrical properties, to tailor their property profile to the applications. The filler content of EMCs for injection molding or transfer molding is generally around 70 wt.% [2,3,4,5,6]. Different kinds of silicate minerals are often used as fillers in EMCs for electronic packaging applications, e.g., kaolin, mica, talc, or micro glass beads, due to their excellent electrical insulation, moisture resistance and a very small coefficient of thermal expansion [7]. Compared to the irregularly shaped particles conventionally used, glass beads have a spherical shape leading to better stress patterns and low local maximum stress levels [8]. Thus, they are widely used as a reinforcement for the plastic industry. In addition, glass beads can be easily and uniformly dispersed throughout the plastic compound and produce smoother flowing compound than with irregular particles [9,10], which ensures that all parts of the electronics are covered with EMC and no short-shot happens even though the filler content is high. In order to improve the bonding between fillers and matrix, the surface of fillers is usually coated with some functional silane as a coupling agent. Altmann et al. observed an increased reaction rate with increasing filler content at low cure temperatures (60–90 °C) and explained this with the catalysis of the epoxy amine reaction by hydrogen donor species present on the silica surface and interfacial effects [11]. Bae et al. also reported similar results based on a liquid-crystalline epoxy resin filled with 5 wt.% of surface-treated carbon nanotubes [12]. Dutta and Ryan compared the reaction kinetics of epoxy compounds filled with 0–10 wt.% surface-treated silica and observed only a decrease of the activation energy but no significant effect on the autocatalyzed reaction with an increase of the filler content [13]. On the other hand, the filler particle size could also affect the reaction kinetics. Wang et al. reported a decrease of the activation energy with the decrease of filler particle size already for a filler content of 0.1 wt.% [14]. However, most research about the influence of fillers on the reaction kinetics is based on relatively low filling contents and the influence of the filler size is also not systematically studied. As mentioned above, the EMCs for electronic packaging applications are generally high-filled. To be able to better control the reactivity and optimize the processing time of EMCs for industrial production, it is necessary to study how filler content and filler size affect the reaction kinetics for high-filled EMCs. 

The aim of this paper is to investigate the influence of a wide range of the filler contents (0–75 wt.%) and filler sizes (*D*_*V*,50_: 8.9–43.2 µm) on the reaction kinetics of an epoxy resin system. The compounds were prepared by a conventional twin-screw-extruder under the same processing condition. The flow-hardening behavior of compounds was investigated with a measuring mixer. Isothermal differential scanning calorimetry (DSC) based on Kamal-Sourour’s model [15] was used to study the reaction kinetics. In addition, a correction of Kamal-Sourour’s model considering the diffusion effect according to Chern and Poehlein [16] was adapted to the calculation of the reaction rate. The influences of filler content and filler size on the kinetic parameters, e.g., the activation energy, reaction order, and diffusion factor, were analyzed and discussed. 

## 2. Isothermal Kinetic Model

Zhao and Hu summarized the kinetic models of thermosets into two main types, (1) nth order and (2) autocatalytic model [17]. The core difference between the two categories of reactions is whether the maximum rate of reaction occurs at zero conversion or at an intermediate conversion value. A simple expression of the nth order model is [18]:

(1) $$\frac{d\alpha }{dt}=k{\left(1-\alpha \right)}^{n}$$

where α is the curing conversion, n is the reaction order, $\frac{d\alpha }{dt}$ is the reaction rate and k is the apparent rate constant. However, Equation (1) is not suitable to describe an auto-catalyzed curing reaction [13,15,18,19], which happens in the epoxy resin reported in this study. Instead, Kamal and Sourour proposed a different model based on the studies of epoxy resins which represents the autocatalytic kinetics [15,19]:

(2) $$\frac{d\alpha }{dt}=k_{1}{\left(1-\alpha \right)}^{n}+k_{2}\alpha^{m}{\left(1-\alpha \right)}^{n}$$

The variable $k_{1}$ describes the rate constant of the nth order reaction, $k_{2}$ describes the rate constant of the autocatalytic reaction with reaction order m [20]. The apparent rate constant $k_{1}$ and $k_{2}$ are expressed in an Arrhenius form:

(3) $$k=A\;\exp\left(-\frac{E_{a}}{RT}\right)$$

where the pre-exponential factor A and the apparent activation energy $E_{a}$ can be determined by linear plotting ln(k) against the inverse of absolute temperature 1/T. R is the universal gas constant. Thus, $E_{a1}$ and $E_{a2}$ describes the activation energy of nth order reaction and autocatalytic reaction, respectively. Equation (2) has been successfully used on the kinetic analysis for many different epoxy systems [12,13,17,18,19,21,22,23].

At the beginning of a curing reaction, the rate of reaction is dominated by the reactivity of the molecules [21]. As the curing progresses, the glass transition temperature (*T_g_*) of thermosets increases. If *T_g_* is well above the curing temperature, the resin will turn from a flexible rubbery state into a rigid glassy state. The reaction will become very slow and diffusion controlled because vitrification is reached. Chern and Poehlein [16] used a semi-empirical equation to describe the diffusion-control in a curing reaction. They assumed that the conversion becomes diffusion-controlled, after a critical value $\alpha_{c}$ is reached. The corresponding conversion rate constant $k_{d}$ is given by:

(4) $$k_{d}=k_{c}e^{\left(-C\left(\alpha -\alpha_{c}\right)\right)}$$

where $k_{c}$ is the rate constant for chemical kinetics and *C* is a constant. 

Equation (4) describes an abrupt change from chemical control into diffusion control after the curing conversion reaches the critical value $\alpha_{c}$. Actually, the change is gradual and there is a region where both controls work together. Chern and Poehlein used the following equation to describe the overall effective rate constant $k_{e}$.

(5) $$\frac{1}{k_{e}}=\frac{1}{k_{d}}+\frac{1}{k_{c}}$$

The diffusion factor f(α) is defined according to Equations (4) and (5) as:

(6) $$f\left(\alpha \right)=\frac{k_{e}}{k_{c}}=\frac{1}{1+e^{\left(-C\left(\alpha -\alpha_{c}\right)\right)}}$$

When the conversion α is much smaller than the critical value $\alpha_{c}$, the term $e^{\left(-C\left(\alpha -\alpha_{c}\right)\right)}$ is almost zero, so that f(α)≈1. In this case, the curing is totally chemical reaction-controlled, and the effect of diffusion is negligible. With the increase of conversion, f(α) is getting smaller than 1. At the point $\alpha =\alpha_{c}$, we have $k_{d}=k_{c}$ and the curing is half reaction-controlled and half diffusion-controlled. Equations (2) and (6) can be united to describe the curing kinetic with the consideration of the diffusion effect:

(7) $$\frac{d\alpha }{dt}=\frac{(k_{1}+k_{2}\alpha^{m}){\left(1-\alpha \right)}^{n}}{1+e^{\left(-C\left(\alpha -\alpha_{c}\right)\right)}}$$

Equation (7) has been successfully used by many authors [17,18,21,22,23] to predict the conversion rate for different kinds of epoxy resins. This equation was also later used to study the reaction kinetics in this paper.

## 3. Experimental

### 3.1. Material

The used thermosetting matrix in this research was an epoxy resin (EP) mixture named Epoxidur 246/1 (Raschig GmbH, Ludwigshafen, Germany). It is a black powder mixture premixed with epoxy resin, hardener, catalyst and some carbon black as a pigment. This EP mixture is used in many EMCs for injection molding and transfer molding. The composition of the mixture is a business secret and hence confidential. The density of the mixture at room temperature is 1.23 g/cm^3^. 

To investigate the influence of fillers on the curing kinetics, A-glass based solid glass beads (Spheriglass^®^, Potters Industriell LLC, Augusta, GA, USA) with different particle sizes were used in this paper. Table 1 shows the manufacturer specifications for the glass beads (GB) used in this paper. The GBs have a coupling agent based on epoxy-silane as standard to enhance the interfacial bonding between GB and EP matrix.

In addition to GBs, a hydrophobic fumed silica (Aerosil^®^ R974, Evonik Resource Efficiency GmbH, Essen, Germany) was used as an additive to thicken the EP compounds, so that the compounds are suitable for injection molding and transfer molding. Since the content of the fumed silica is much less than the content of GBs and the influence of fumed silica is not the purpose of this paper, the content of the fumed silica was kept constant at 5 wt.% in all formulations.

### 3.2. Sample Preparation

The compounds used in this paper were produced with a twin-screw-extruder. Firstly, the EP mixture, fumed silica and GBs were premixed together according to the recipe in Table 2. In formulation A, B, and C, the same filler GB3000 was used and the filler content varies from 40 to 70 wt.%. In formulation B, D, and E, different fillers were used but the filler content remains the same at 55 wt.%. Compared to formulation B, formulation B* without R974 was additionally prepared to investigate the effect of R974 on the ultimate glass transition temperature.

**Figure 1:**
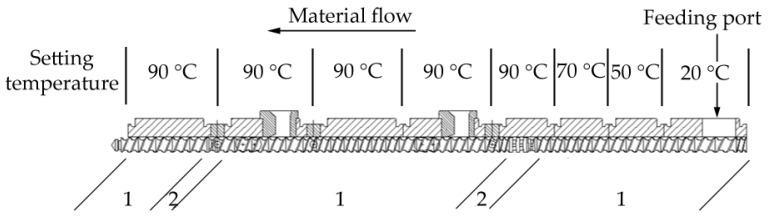
Used screw configuration and setting the temperature of heating blocks on the twin-screw-extruder (TSE) for the preparation of compounds (1. conveying elements, 2. kneading elements).

The mixture of all components was fed into a twin-screw-extruder (ZSE 25Ax45D, KraussMaffei Berstorff GmbH, Hannover, Germany) with a screw diameter of D = 25 mm and a screw length of L = 45.5 D. The processing temperature of all cylinder blocks are described in Figure 1. Due to the high thermal sensitivity of epoxy resins, only two groups of kneading elements were used for processing. The maximal temperature zone was set up to 90 °C, so that the EP is molten but no curing takes place. After the mixture was extruded as strings, it was cooled using a vibration chiller and further granulated with a rotation crusher in order to form suitable pellets for e.g., injection molding or transfer molding. The pellets were used as samples for the following rheological and thermal analysis. 

### 3.3. Testing Methods

To characterize the particle size distribution of used GBs, an optical camera with static image analysis (Morphologi G3s, Malvern Panalytical GmbH, Kassel, Germany) was used. As the GB particles are spherical, the equivalent diameter of the sphere was used as the measurement index for the particle size distribution. The volume of measured GB samples was 3 mm^3^. Up to 100,000 particles were counted in a measurement. 

**Figure 2:**
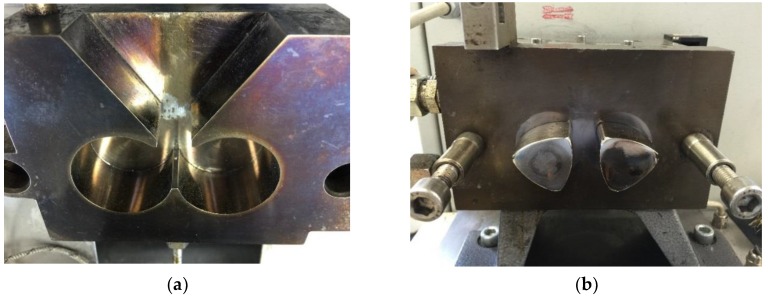
Pictures of measuring mixer (**a**) mixer bowl with an opening on the top to fill testing material (**b**) mixing blades on the rear site.

**Figure 3:**
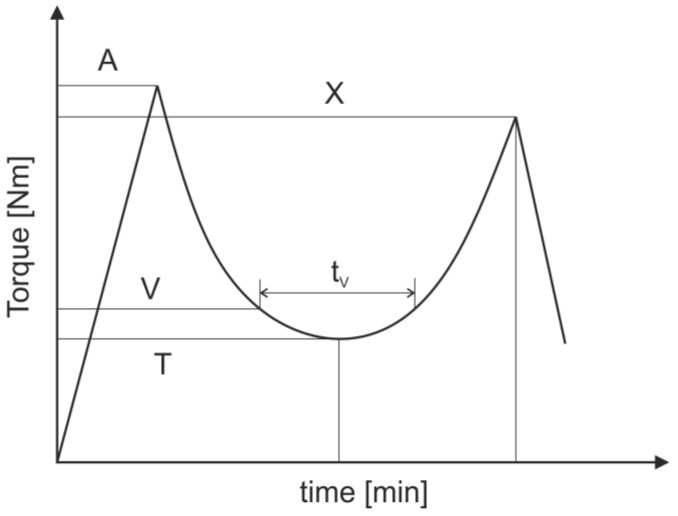
Interpretation of the temperature-dependent flow and hardening behavior measured closely aligned with the German standard DIN 53764 (presently withdrawn) and the recommendations of Brabender GmbH Co. & KG. A: Loading peak, *T*: Minimum torque, *X*: Maximum torque after curing, *V*: Residence torque calculated to *B* + 10% of the difference between *X* and *B*, *t_V_*: residence time.

A measuring mixer (Mixer MB30, Brabender GmbH & Co. KG, Duisburg, Germany) as shown in Figure 2 was used as a rheometer to characterize the temperature-dependent flow and hardening behavior of the compounds. The testing material is loaded at 20 °C through the top opening into the heated mixer bowl where it is homogenized by specially shaped mixing blades. The required torque to maintain the constant rotation speed of 30 rpm was measured in the test. As the curing progresses further, the viscosity of the testing material increases and so does the needed torque. At the point of time, when the testing material can no longer be viscously deformed, it will be ground into small pieces. Thus, the torque reaches a peak point *X* and drops after that. Since the needed torque depends on the viscosity of the compounds, the minimum torque *T* and resident time *t_V_* were analyzed as an index for the flow ability and processing time for the testing compound under the testing condition. The value *T* depends on the minimum viscosity of the testing material. The value *t_V_* signifies, how long the material is able to be shaped under the tested temperature. The experiment was carried out with a mixer temperature of 110 °C and a sample size of 18.75 cm^3^, which is 75 vol.% of the total bowl space. Under this testing temperature, the difference of the minimum torque *T* and resident time *t_V_* between different compounds can be measured clearly. Figure 3 shows a typical curve of a thermosetting molding compound. The residence time *t_V_* is defined as the duration for which the torque is in the range of *T* to *T* + 0.1 × (*X* − *T*). 

**Figure 4:**
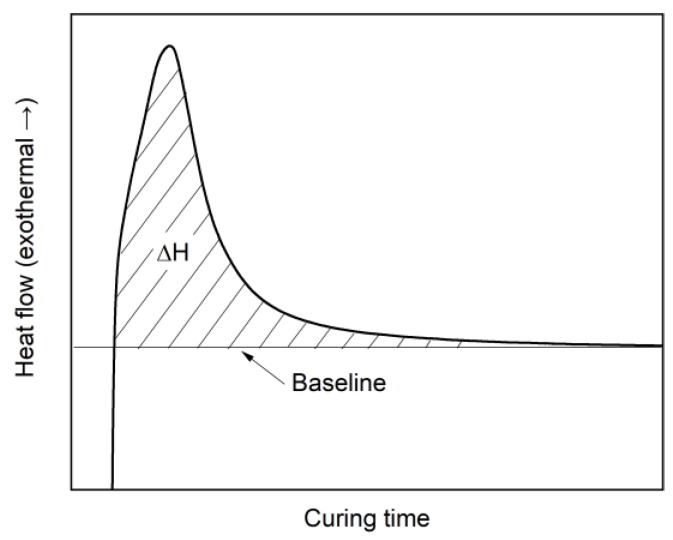
Determination of $∆H_{iso}$ in a schematical isothermal differential scanning calorimetry (DSC) curing curve.

To study the reaction kinetics of different formulations, isothermal calorimetric measurements with a DSC (Discovery DSC2A, TA Instruments Inc., New Castle, Delaware, USA) were carried out at different temperatures (120 °C, 130 °C, 140 °C, 145 °C, and 155 °C) for every formulation. The instrument was calibrated with indium and zinc standards. The curing conversion α was calculated as

(8) $$\alpha =\frac{∆H_{total}-∆H_{iso}}{∆H_{total}}$$

where $∆H_{total}$ is the total heat of the curing reaction which was determined by a separate temperature-sweep DSC with 2 K/min from 20 °C to 240 °C, $∆H_{iso}$ is the reaction heat during the isothermal holding process, which is given by the area above the baseline and below the curve as shown in Figure 4. The baseline is a straight tangential line to the horizontal part of the isothermal DSC curve. For every material, $∆H_{total}$ was measured 3 times and the mean value was used for the calculation of the curing conversion. After the first heating step, samples were quickly cooled down to 20 °C and heated with 20 K/min to 240 °C to investigate the ultimate glass transition temperature. 

**Figure 5:**
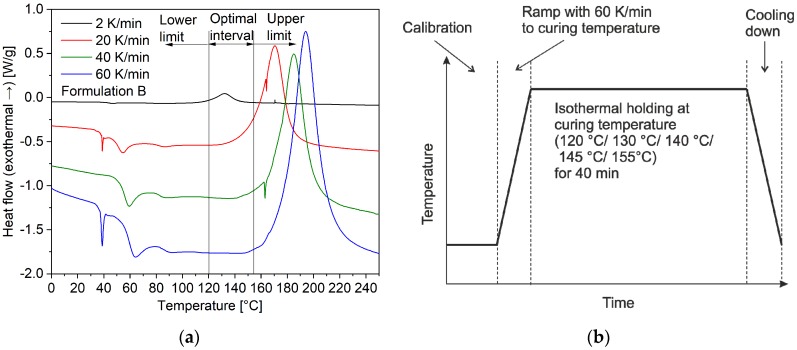
(**a**) The optimal interval of isothermal holding temperature (120–155 °C) was determined by several dynamical DSC measurements, (**b**) Isothermal DSC measuring program.

Salla and Ramis [24] compared different methods to calculate the curing conversion based on an unsaturated polyester using isothermal DSC measurements, including the method used in this paper. They pointed out that one disadvantage of this method is that $∆H_{iso}$ could be not correct because of part of the $∆H_{iso}$ cannot be registered by the calorimeter at the beginning and at the end of the reaction. To avoid that, it is important to use a relatively high heating rate to reach the isothermal temperature, and meanwhile, no curing begins before the holding temperature arrives. Thus, the range of isothermal temperature should be determined firstly by several dynamical DSC for formulation B. Samples (5–10 mg) were prepared directly in DSC aluminum pans with a lid and heated at different rates (2, 20, 40 and 60 K/min) from 20 °C to 240 °C. The experiments were carried out in nitrogen at a flow rate of 50 cc/min. Figure 5a shows the results of the measurements. At the lowest heating rate (2 K/min), the curing begins at 120 °C (onset point). If the isothermal holding temperature is under 120 °C, the heat flow signal has a lot of noise, as the curing rate is slow. At the highest heating rate (60 K/min) the curing reaction begins at 155 °C (onset temperature). To make sure, the reaction heat at the beginning of the isothermal holding step is also detected, the isothermal holding temperature should be limited to 155 °C. The optimal interval isothermal temperature was hence selected from 120 °C to 155 °C. A heating rate of 60 °C/min was used to reach the isothermal temperatures. Isothermal experiments were carried out at 120 °C, 130 °C, 140 °C, 145 °C, and 155 °C. A graphical representation of the isothermal DSC measuring program is shown in Figure 5b. Nitrogen gas was used for purging at a flow rate of 50 cc/min. The isothermal holding step is stopped, after no change of the heat flow can be detected. 

## 4. Results

### 4.1. Characterization of Fillers and Compounds

**Figure 6:**
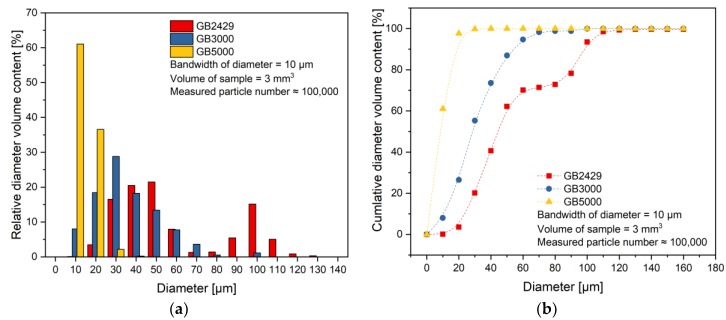
(**a**) Volume-based diameter distribution of glass beads (GB) at 10 µm bandwidth (**b**) Cumulative volume content of GBs based on their diameter.

The particle size distribution based on volume content for used GBs is shown in Figure 6 a,b. In Figure 6a, the relative diameter volume contents were calculated at every 10 µm bandwidth. The diameter of GB5000 particles is mostly around 0–20 µm. Compared to that, GB3000 particles have a wider range of diameter distribution, mostly between 10 and 60 µm. GB2429 shows two peaks in the diameter distribution, one at around 40–60 µm, another one at 100 µm. In Figure 6b, the cumulative diameter volume content was calculated with increasing diameter bands, showing clearly that GB2429 has the highest average particle size of all three fillers and GB5000 has the smallest particles. Table 3 summarizes the results of the particle size distribution with *D*_*V*,10_, *D*_*V*,50_, and *D*_*V*,90_. A value of *D*_*V*,50_ = 8.89 µm for GB5000 signifies that 50 vol.% of the GB 5000 particles are smaller than 8.89 µm. 

**Figure 7:**
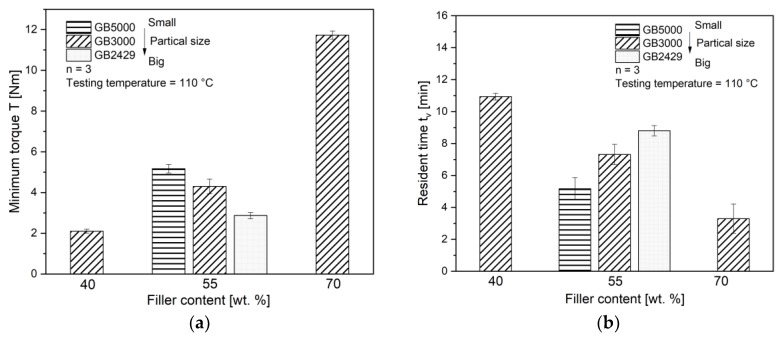
The measured (**a**) minimum torque *T* and (**b**) resident time *t_v_* of compounds.

The temperature-dependent flow-hardening behavior was quantified with the measuring mixer. As shown in Figure 7a, the minimum torque *T* increases with increasing filler content. The value *T* of 70 wt.% filled compounds is almost 6 times higher than that of 40 wt.% filled compounds. For compounds with the same filler content of 55 wt.% but different filler size, the minimum torque *T* decreases with increasing filler size. However, the influence of the filler content dominates over the influence of the filler size on the minimum torque *T*. The value *T* of the compounds with the smallest filler is only about 2 times higher than that with the biggest filler. On the other side, the increase of filler content has an adverse impact on the resident time *t_v_*. The value *t_v_* reduces from 11 min for 40 wt.% filled compounds to about 3 min for 70 wt.% filled compounds. Compared to that, the increase of filler size has a positive impact on value *t_v_*. The value *t_v_* of the compounds with the biggest filler GB2429 is about 9 min, which is almost 2 times higher than *t_v_* of the smallest filler GB5000. Similar to the value *T*, the filler content shows here greater influence on the value *t_v_* than the filler size. As the resident time *t_v_* correlates with the possible processing time of the material, the increase of filler content and reduction of filler size lead to a tight processing window for the shaping. To understand how this happens, the curing kinetics of the different compounds were studied with isothermal DSC measurements.

**Figure 8:**
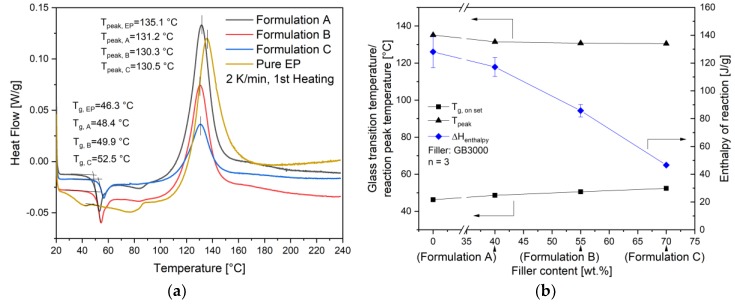
(**a**) The representative DSC dynamic measurement curves of formulations with the same filler size, but different filler contents, (**b**) Influence of filler content on the glass transition temperature *T_g_*, the reaction peak temperature *T_peak_* and the enthalpy of reaction *ΔH_total_* of compounds.

**Figure 9:**
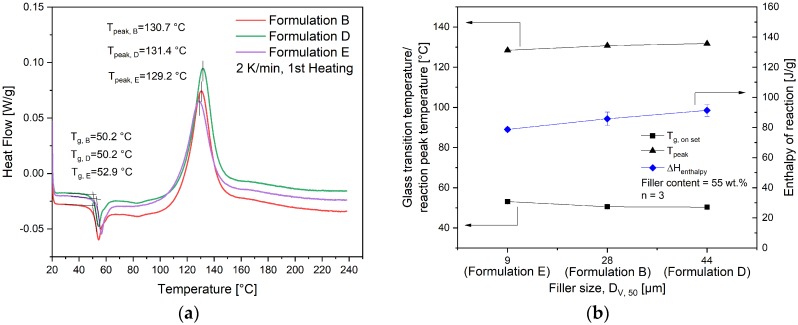
(**a**) The representative DSC dynamic measurement curves of formulations with the same filler content 55 wt.%, but different filler sizes (**b**) Influence of filler size on the glass transition temperature *T_g_*, the reaction peak temperature *T_peak_* and the enthalpy of reaction *Δ*H_total_ of compounds.

As shown in Equations (7) and (8), to calculate the kinetic parameters, the total reaction enthalpy $∆H_{total}$ of the compounds must be known. It was measured with a temperature sweep DSC with a heating rate of 2 K/min from 20 °C to 240 °C. The representative curves of pure EP and compounds filled with the same filler GB3000 but different filler content are compared in Figure 8a. The glass transition temperature *T_g_* is evaluated as the onset point of the curve. Though all the compounds were manufactured under the same compounding parameters, the *T_g_* of granulates shows a slightly increasing trend with the increasing filler content as summarized in Figure 8b. Considering that the *T_g_* increases with increasing curing degree [25], the result indicates that the epoxy resin in high-filled compounds is slightly more cured. As the shear heating between fillers increases with the increasing filler content, the melt temperature of high-filled compounds could be higher than the setting temperature of the heating blocks. This could cause more curing in the EP resin. The exothermal peak temperatures *T_peak_* of all three compounds are around 130 °C, which is lower than the pure EP (135.1 °C). The total reaction enthalpy $∆H_{total}$ decreases with the increasing filler content, because the GB itself is not reactive. On the other hand, the curves of formulations with the same filler content of 55 wt.% but different filler sizes were compared in Figure 9a. The *T_g_* of formulation E, which contains the smallest filler, is approximately 2.5 K higher compared to the other two materials. This indicates a slightly more cured epoxy resin in this material. Meanwhile, the *T_peak_* of formulation E is also the lowest in all three compounds as summarized in Figure 9b. The total reaction enthalpy $∆H_{total}$ increases with the increasing particle size even though these materials have an identical filler content, which indicates that the compound with smaller filler particles is more cured in the compounding process. 

**Figure 10:**
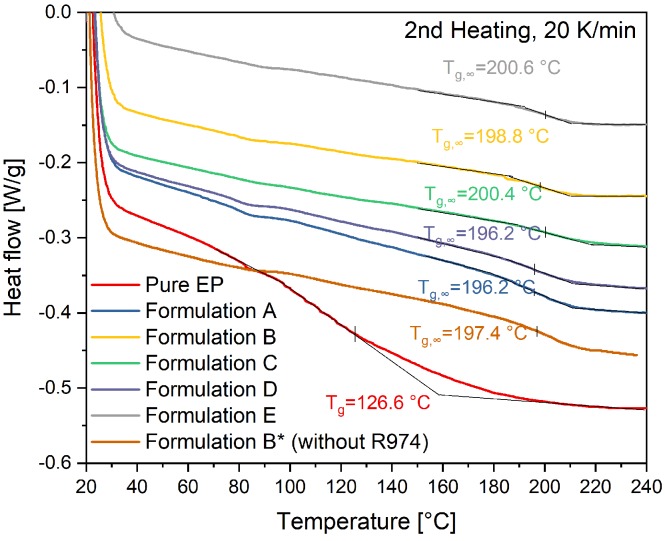
Determination of the ultimate glass transition temperature with the half-height method in the 2nd heating of temperature-sweep DSC.

The ultimate glass transition temperature (*T_g,∞_*) is evaluated by the half-height method in the 2nd heating of temperature-sweep DSC in Figure 10. The *T_g,∞_* of all glass-bead-filled compounds, is around 198 °C. It is hard to evaluate the *T_g,∞_* of pure EP with the half-height method because the base-lines are difficult to be defined. Nevertheless, the *T_g,∞_* of pure EP is comparatively significant lower than other compounds. The *T_g,∞_* of pure EP could be around 126 °C. Additionally, in comparison to formulation B, formulation B* without R974 has almost the same *T_g,∞_*. This result indicates that R974 has barely any influence on the *T_g,∞_*. The surface-treated glass beads lead to the significant increase of the *T_g,∞_*. Possibly, the epoxy-silane-based coupling agent on the surface of glass beads could react with EP resins. Thus, the reaction mechanism of pure EP might be quite different from filled compounds. The reaction kinetics should be therefore studied and compared. 

### 4.2. Calculation of Autocatalytic Kinetic Model Parameters

**Figure 11:**
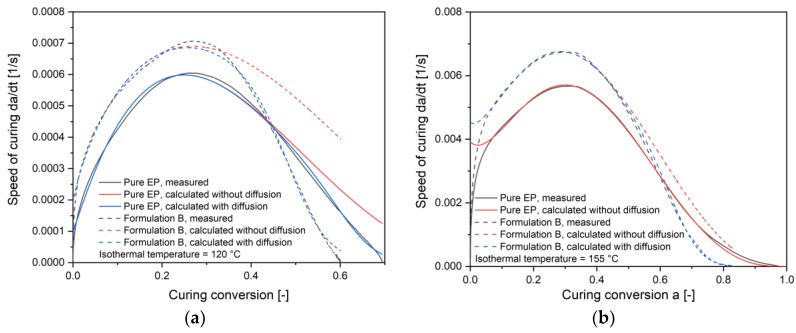
Measured curing speed in comparison to calculated speed with and without the diffusion factor at isothermal temperature (**a**) 120 °C and (**b**) 155 °C.

The reaction kinetics of the compounds were studied by using Kamal-Sourour’s model with the adjustment of the diffusion factor as presented in Equation (7). To generate the kinetic parameters (*k*_1_, *k*_2_, *m*, *n*, $\alpha_{c}$, *c*), five isothermal DSC experiments at different temperatures were carried out for each material. The Kamal-Sourour’s model without the diffusion factor is firstly used to fit the experimental data until the coefficient of determination *R*^2^ is less than 0.99. After that, the diffusion factor was used for the further correlation of a nonlinear fit to the measured data. All the fitted kinetic parameters are listed in Table 4. For pure EP, the modification of Kamal-Sourour’s model with the diffusion factor is needed only for relatively low temperatures 120 °C, 130 °C, and 140 °C. Figure 11a shows the fitted curves with and without the diffusion factor at 120 °C as an example. Similar to pure EP, formulation B also needs the modification of the diffusion factor at this temperature. The calculated curing speed without the diffusion factor is much higher than the measured value for the curing conversion a > 0.3. At higher isothermal temperatures, for example, 155 °C in Figure 11b, the calculation of the curing speed without the diffusion factor fits already well for pure EP. However, for all the filled compounds, the modification with the diffusion factor is still needed. Meanwhile, the difference between the calculated curing speed with and without the diffusion modification is getting smaller with increasing isothermal temperature. The reason for this phenomenon could be the increased agility of monomers and polymer chains at a higher temperature so that the barrier of diffusion is not reduced. Furthermore, Fraga et al. [26] explained, when a thermosetting system is cured at a temperature higher than its ultimate glass transition temperature, diffusion phenomena may not affect the kinetics of reaction. 

As shown previously in Figure 10, the ultimate glass transition temperature of pure EP is around 126.6 °C. The vitrification of pure EP will not happen when the curing temperature is higher than the ultimate glass transition temperature. That matches well with the fact that when the curing temperature is above 140 °C, no diffusion modification to Kamal-Sourour’s model is needed to fit the measured curing speed. On the other side, the ultimate glass transition temperature of all the filled compounds is around 200 °C and much higher than the maximal curing temperature. As the glass transition temperature of compounds increases continuously with increasing curing degree and finally surpasses the isothermal curing temperature, the vitrification will take place. In this case, the agility of polymer chains will be strongly limited. The curing process turns in to a diffusion-controlled process instead of reaction controlled. That matches well with the fact that all the filled compounds need diffusion modification to fit the measured curing speed in the later conversion. 

### 4.3. Influence of Filler Content on the Curing Kinetics

**Figure 12:**
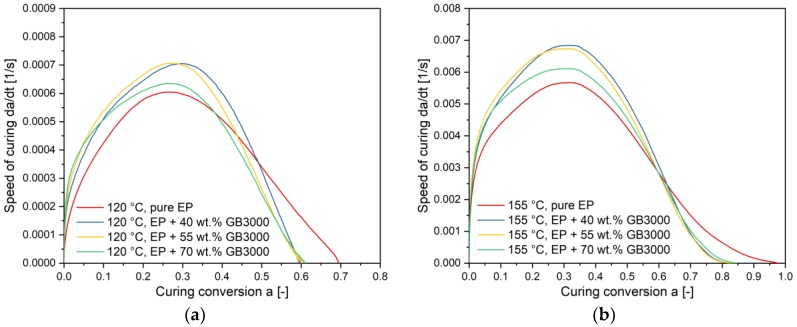
Curing speed versus curing conversion for compounds with different filler content at (**a**) 120 °C and (**b**) 155 °C.

**Figure 13:**
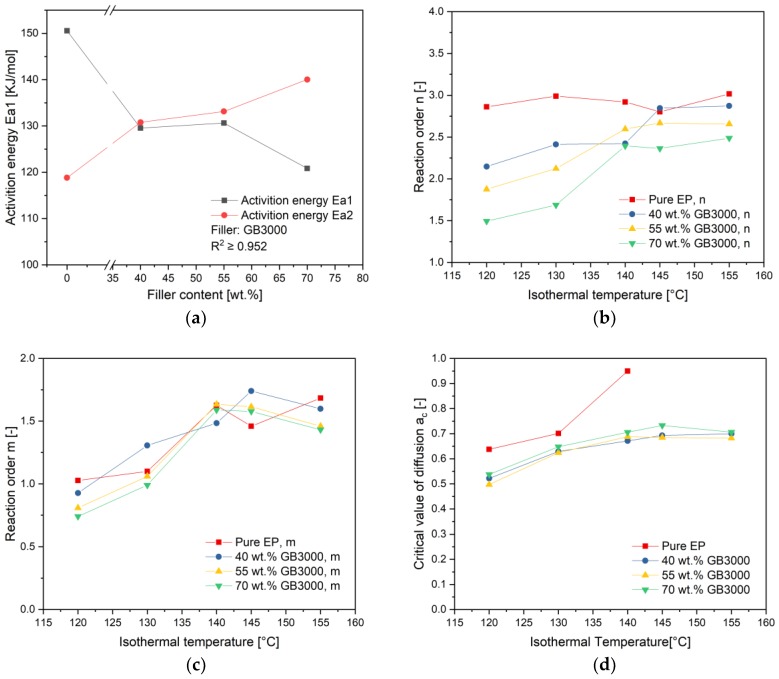
Influence of filler content on (**a**) activation energy (**b**) reaction order n for the nth order reaction (**c**) reaction order m for the autocatalytic reaction (**d**) critical value of diffusion *a_c_*.

To analyze the influence of filler content to curing kinetics, the curing speed curves versus conversion of compounds with the same filler size but different content is described at the curing temperature 120 °C and 155 °C as an example in Figure 12a,b. At 120 °C, the curing speed of pure EP, in the beginning, is lower than of the compounds with fillers. However, in the later curing stage, the curing speed of compounds with fillers reduces more quickly, especially after the peak point. The compound with the highest filler content has the highest curing speed in the beginning but also reduces at the fastest rate in all three variants. Since the ultimate *T_g_* of pure EP is lower than those of filled compounds as shown previously, its curing conversion reaches a higher final value. The same phenomenon was also observed for all the other isothermal temperatures. Figure 12b shows, for example, the curing speed versus curing conversion at 155 °C. As the curing conversion goes over 0.1, the curing speed of the 70 wt.% filled compounds drops already behind the other filled compounds. The curing of pure EP starts with the lowest speed but surpasses the filled compounds after the conversion rises above 0.6. A possible explanation for the quickly reduced curing speed of high-filled compounds can be given on the basis of the increasing barriers between thermoset resins. As the curing going up, the polymer chains grow up and the movement space of oligomers reduces simultaneously. For high-filled compounds, the movement space of monomers is even smaller. Thus, the curing speed of high-filled compounds drops more quickly. For further analyzation of the results, the reaction parameters were compared in Figure 13.

Figure 13a shows the activation energy $E_{a1}$ and $E_{a2}$ in dependency of filler content. As explained previously, $E_{a1}$ and $E_{a2}$ correspond to the activation energy of the nth order reaction and the autocatalytic reaction, respectively. The value of $E_{a1}$ has a decreasing trend with the increasing filler content, while $E_{a2}$ has an inverse trend. The Kamal-Sourour’s model can be also described as follow:

(9) $$\frac{d\alpha }{dt}=\left(k_{1}+k_{2}\alpha^{m}\right){\left(1-\alpha \right)}^{n}$$

Since, at the beginning of curing reaction α is almost zero, the term $k_{2}\alpha^{m}$ is also almost zero. Thus, the reaction speed is mostly dependent on the nth order reaction in the beginning. A material with lower activation energy needs less energy to kick off the reaction. In other words, under the same temperature, the material with lower activation energy has a faster curing speed. That explains why the curing speed increases with filler content in the beginning. For another, the value $E_{a2}$ increases with filler content, which suggests the autocatalytic mechanism win more places at a higher temperature in the total curing kinetic. Some researchers [13,18] reported surface-treated silica fillers can accelerate the curing reaction of epoxy resin. Hendra et al. [27] observed an acceleration effect of epoxy resin with the increasing filler content for untreated fillers. Thus, the results reported in this paper could be the overlaying of these two effects.

On the basis of Table 4, the reaction order *n* and *m* versus the temperature of compounds with different filler contents have been described in Figure 13b,c, respectively. As can be seen in Figure 13b, the reaction order *n* of pure resin remains at about 3 unchanged to temperature. While the value *n* of filled compounds trends to increase with temperature and stabilize finally to a value under 3. Compounds with more filler content show here lower values of *n* at a certain curing temperature. Generally speaking, a greater value of *n* is attributed to the trimolecular mechanism [28]. The fact, that the value of *n* decreases with increasing filling content, can be explained with the glass beads inhibiting the crosslinking of epoxy resin in three-dimensional direction because spaces for reaction of resins were taken by glass beads. As the curing temperature rises, the movement of the monomers increases and the inhibition effect of glass beads decreases. Thus, the reaction order n increases with temperature. On the other side, the reaction order *m* of pure EP and filled compounds presents an increasing trend to increasing curing temperature as shown in Figure 13c. A clear dependence between the value of *m* and filler content cannot be observed. As previously explained, the value of *m* represents the reaction order of the autocatalytic reaction. The increased value *m* with temperature means that the autocatalytic reaction has a higher share in the overall reaction mechanism. 

Figure 13d shows the influence of filler content on the critical value of diffusion *a_c_* at different temperatures. It can be seen clearly that the value *a_c_* increases with curing temperature. Note that in the vicinity of vitrification, the curing reaction becomes diffusion-controlled, because the movement of polymer chains is severely restricted. The value *a_c_* is subsequently reached at the vitrification point when *T_g_* reaches the curing temperature [26]. Thus, the value *a_c_* increases with the curing temperature as long as the curing temperature is lower than the ultimate *T_g_*. For pure EP, its *a_c_* is generally higher than the one of the filled compounds, because its ultimate *T_g_* is much lower than those of filled compounds. When the curing temperature is higher than the ultimate *T_g_* of pure EP (126.6 °C), the vitrification will not initiate, the modification of Kamal-Sourour’s model with the diffusion factor is therefore not needed. On the other hand, a clear dependence of the value *a_c_* to the filler content cannot be observed.

### 4.4. Influence of Filler Content on the Curing Kinetics

**Figure 14:**
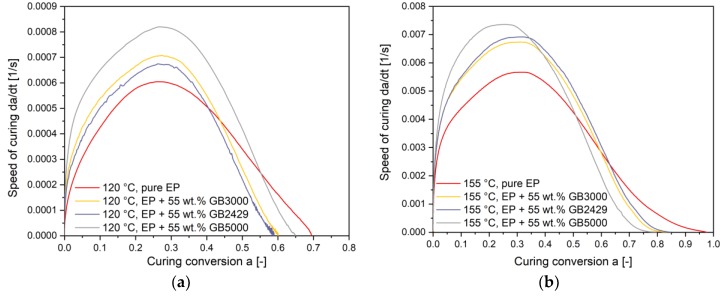
Curing speed versus curing conversion for compounds with different filler size at (**a**) 120 °C and (**b**) 155 °C.

**Figure 15:**
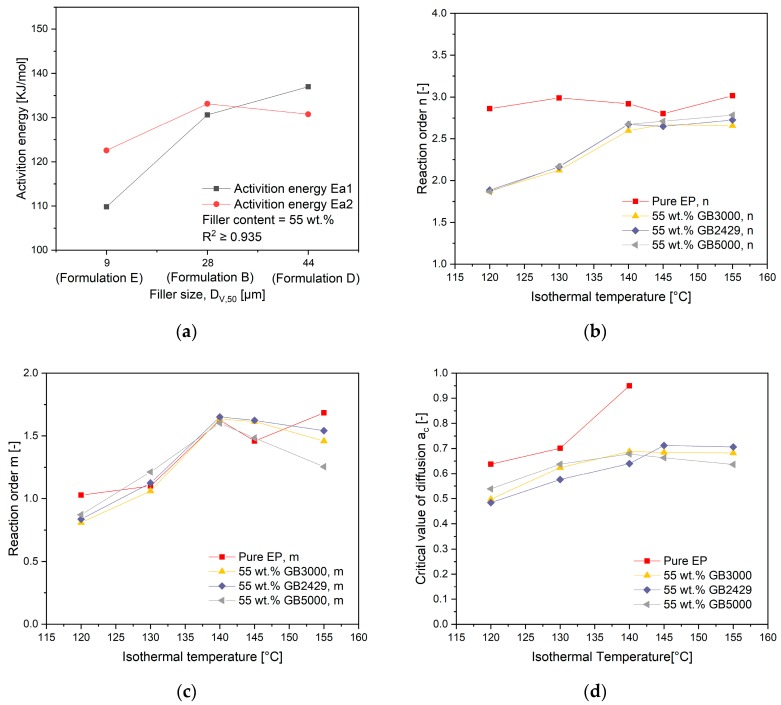
Influence of filler size on (**a**) activation energy (**b**) reaction order *n* for the nth order reaction (**c**) reaction order *m* for the autocatalytic reaction (**d**) critical value of diffusion *a_c_*.

Figure 14 shows the influence of filler size on the curing speed for (a) 120 °C and (b) 155 °C. As can be seen, at 120 °C, compounds with a smaller filler size have generally faster curing speeds over the total conversion. However, the curing speed of pure EP surpasses the filled compounds later because the used curing temperature is closer to its ultimate *T_g_*. At 155 °C, the curing speed of the compounds with the smallest filler is the fastest in the beginning but reduces more rapidly after its peak point than the other filled compounds. To explain the results, the reaction kinetic parameters were analyzed in Figure 15.

Figure 15a shows a general decrease in the activation energy $E_{a1}$ and $E_{a2}$ with the increasing filler size. Especially, the activation energy $E_{a1}$ of the compounds with the smallest filler is over 18% lower than in the other compounds, which explains its fastest curing speed at the beginning of curing reaction. Meanwhile, the activation energy $E_{a2}$ is also the smallest for the compounds with the smallest filler. For another, the reaction order *n* indicates no dependency on the used filler size as shown in Figure 15b. Only an increase of the value n with rising curing temperature is observed, as previously stated. Interesting here is the change of the reaction order *m* in relation to the temperature in Figure 15c. While filled compounds show generally an increase of the value *m* with rising temperature and stabilize finally to a certain value, the compounds with the smallest filler have a clear decrease in a high-temperature range. As can be seen in Equation (2), when the conversion progresses further, the influence of the nth order reaction term $k_{1}{\left(1-\alpha \right)}^{n}$ is getting smaller and the contribution of the autocatalytic reaction term $k_{2}\alpha^{m}{\left(1-\alpha \right)}^{n}$ to the total reaction speed is getting more and more. A smaller value of m for the compounds with the smallest fillers at high temperature means its autocatalytic reaction part is slower than in the other compounds, which explains its slower reaction speed in Figure 1b after a conversion about 0.4. As can be seen in Figure 15d, the critical value of diffusion *a_c_* shows a temperature dependency. No clear dependence of the value a_c_ to the used filler size can be found in Figure 15d. 

## 5. Conclusions

In this article, the influences of content and particle size of surface-treated fillers on the flow-hardening behavior of epoxy resin-based compounds were investigated firstly by using a measuring mixer. Later, to understand the rheological results, the reaction kinetics were studied with isothermal DSC measurements. A classical kinetic model Kamal-Sourour’s model was successfully adapted to fit the measured data for the early stage conversion. To fit the later stage conversion, an adjustment of Kamal-Sourour’s model with the diffusion factor according to Chern and Poehlein was needed, if the ultimate *T_g_* of the testing material is higher than the curing temperature, which happens on all the filled compounds. As the ultimate *T_g,∞_* of pure EP is about 126.6 °C, Kamal-Sourour’s model can fit the measured data well for a curing temperature above 140 °C without the adjustment of diffusion. Furthermore, the influence of filler content and size to reaction speed and kinetic parameters were analyzed and discussed. Compounds with higher filler content and smaller filler size show lower activation energy $E_{a1}$, which results in a faster curing speed at the beginning of the curing reaction. As the curing progresses, the curing speeds of compounds with higher filler content or smaller filler size reduce more quickly. The smaller reaction order *n* of the higher filled compounds should be responsible for the rapid decrease of curing speed. A possible reason for that is the more limited movement space of polymer chains and monomers in higher filled compounds, so the nth order reaction is limited. A smaller reaction order m was observed for compounds with smaller filler size, which leads to a quick reduction of curing speed in the later time of curing. The critical diffusion value *a_c_* of all the filled compounds increases quickly in the low curing temperature range and stabilize around 0.6 because the used curing temperature is still below the ultimate *T_g,∞_*. A clear dependence of filler content and filler size to the critical diffusion value a_c_ cannot be identified. 

In conclusion, the content and particle size of surface-treated glass beads used in this research have a significant influence on the curing speed and reaction kinetics. The acceleration effect of an increased filler content or a reduced filler size on the reaction speed must be considered in manufacturing.

## Figures and Tables

**Table 1:** Particle size and bulk density of the used glass beads as specified by the manufacturer.

Product	Particle Size Distribution * [µm]		Bulk Density [g/cm^3^] ASTM D-3101-78	
	Mean Value	Range	Untapped	Tapped
GB2429	70–100	53–106	1.46	1.57
GB3000	30–50	-	1.44	1.59
GB5000	7–10	-	1.28	1.62

*: typical values.

**Table 2:** Overview of components used in different formulations.

Formulation	Polymer Matrix	Additive and Content	Filler and Content
A	EP 246-1	R974, 5 wt.%	GK3000, 40 wt.%
B	EP 246-1	R974, 5 wt.%	GK3000, 55 wt.%
C	EP 246-1	R974, 5 wt.%	GK3000, 70 wt.%
D	EP 246-1	R974, 5 wt.%	GK2429, 55 wt.%
E	EP 246-1	R974, 5 wt.%	GK5000, 55 wt.%
B*	EP 246-1	-	GK3000, 55 wt.%

**Table 3:** Summary of the particle distribution of used GBs.

Filler	Diameter [µm]		
	*D*_*V*,10_	*D*_*V*,50_	*D*_*V*,90_
Spheriglass GB5000	4.77	8.89	15.75
Spheriglass GB3000	10.98	25.84	56.26
Spheriglass GB2429	23.78	43.22	96.87

**Table 4:** Parameters used in Kamal-Sourour’s model with diffusion factor (Equation (6)).

Parameter	Temperature [°C]	Pure EP	EP + 40 wt.% GB3000	EP + 55 wt.% GB2429	EP + 55 wt.% GB3000	EP + 55 wt.% GB5000	EP + 70 wt.% GB3000
**k1**	120	9.758 × 10^−5^	1.749 × 10^−4^	1.661 × 10^−4^	1.878 × 10^−4^	3.268 × 10^−4^	2.312 × 10^−4^
**k2**		5.260 × 10^−3^	3.950 × 10^−3^	3.070 × 10^−3^	3.050 × 10^−3^	3.510 × 10^−3^	2.210 × 10^−3^
**m**		1.027	0.928	0.836	0.809	0.871	0.740
**n**		2.862	2.147	1.887	1.875	1.867	1.493
**A**		0.638	0.522	0.484	0.497	0.539	0.537
**C**		22.707	38.189	27.170	21.550	20.532	21.921
**R^2^**		0.985	0.990	0.992	0.997	0.996	0.993
**k1**	130	3.082 × 10^−4^	6.115 × 10^−4^	5.363 × 10^−4^	5.422 × 10^−4^	8.630 × 10^−4^	6.522 × 10^−4^
**k2**		1.035 × 10^−2^	1.398 × 10^−2^	9.250 × 10^−3^	9.520 × 10^−3^	1.183 × 10^−2^	6.580 × 10^−3^
**m**		1.100	1.306	1.124	1.060	1.213	0.990
**n**		2.990	2.413	2.163	2.123	2.169	1.688
**Ac**		0.701	0.629	0.576	0.624	0.638	0.648
**C**		22.594	20.759	20.374	21.485	23.270	20.313
**R^2^**		0.992	0.994	0.997	0.996	0.995	0.996
**k1**	140	1.260 × 10^−3^	1.440 × 10^−3^	1.620 × 10^−3^	1.780 × 10^−3^	2.210 × 10^−3^	1.830 × 10^−3^
**k2**		4.239 × 10^−2^	2.732 × 10^−2^	3.723 × 10^−2^	3.936 × 10^−2^	4.125 × 10^−2^	2.994 × 10^−2^
**m**		1.626	1.484	1.650	1.635	1.603	1.589
**n**		2.920	2.423	2.671	2.596	2.675	2.394
**Ac**		0.950	0.671	0.640	0.688	0.678	0.705
**C**		10.390	21.060	25.584	19.635	20.854	25.008
**R^2^**		0.900	0.995	0.993	0.996	0.995	0.990
**k1**	145	1.640 × 10^−3^	2.440 × 10^−3^	2.440 × 10^−3^	2.480 × 10^−3^	2.87 × 10^−3^	2.650 × 10^−3^
**k2**		3.707 × 10^−2^	6.220 × 10^−2^	5.461 × 10^−2^	5.201 × 10^−2^	4.905 × 10^−2^	4.126 × 10^−2^
**m**		1.459	1.740	1.624	1.615	1.484	1.577
**n**		2.802	2.846	2.647	2.668	2.712	2.364
**Ac**		*	0.693	0.712	0.685	0.663	0.733
**C**		*	17.680	23.780	23.120	24.660	26.267
**R^2^**		*	0.988	0.994	0.993	0.990	0.980
**k1**	155	3.920 × 10^−3^	4.390 × 10^−3^	4.850 × 10^−3^	4.520 × 10^−3^	4.860 × 10^−3^	4.370 × 10^−3^
**k2**		9.715 × 10^−2^	1.008 × 10^−1^	8.653 × 10^−2^	7.468 × 10^−2^	6.612 × 10^−2^	5.883 × 10^−2^
**m**		1.683	1.598	1.542	1.460	1.255	1.431
**n**		3.016	2.873	2.727	2.656	2.785	2.488
**Ac**		*	0.699	0.706	0.683	0.637	0.706
**C**		*	25.485	22.040	21.204	24.391	21.266
**R^2^**		*	0.993	0.990	0.994	0.993	0.990

*: no need for the diffusion factor.
